# Expanding the role of robotic surgery: Non-inferiority of robot-assisted radical cystectomy in overweight patients with bladder cancer

**DOI:** 10.1007/s11701-025-02939-5

**Published:** 2025-11-12

**Authors:** Jakob Kohler, Leonhard Buck, Konrad Hügelmann, Reha-Baris Incesu, Hans Christoph von Knobloch, Tim Krumm, Julian Risch, Patricia Schließer, Oscar Weische, Marie Weiss, Jonas Jarczyk, Severin Rodler, Philipp Nuhn

**Affiliations:** 1 Department of Urology, University of Kiel (UKSH), Arnold-Heller-Strasse 1-3, 24105 Kiel, Germany; 2 Kurt-Semm Center for laparoscopic and robotic surgery, University Hospital Schleswig-Holstein Campus Kiel, Kiel, Germany

**Keywords:** Robot-assisted radical cystectomy, Open radical cystectomy, Bladder cancer, Obesity, Complications

## Abstract

**Background:**

Obesity is an increasing comorbidity in patients undergoing radical cystectomy. While robot-assisted radical cystectomy (RARC) has been widely adopted, evidence for its safety in overweight patients remains limited.

**Methods:**

We retrospectively analyzed 337 consecutive patients who underwent RARC or open radical cystectomy (ORC) at a European tertiary center (2013–2024). Outcomes were stratified by body mass index (BMI) and assessed by regression and non-inferiority analyses.

**Results:**

In the presented cohort (135 normal-weight and 202 overweight patients) urinary-diversion patterns (continent vs. incontinent) did not differ by BMI or approach. RARC required longer operative times than ORC, but BMI itself had no independent effect. The length of stay (LOS) was shorter after RARC with BMI increasing LOS only in the ORC group. Adjusted models identified ORC as an independent predictor of prolonged stay. Non-inferiority of RARC in overweight patients was demonstrated for both length of stay (*p* = 0.044) and Clavien–Dindo-graded perioperative morbidity.

**Conclusion:**

RARC delivers robust perioperative safety in overweight patients, maintaining non-inferiority for morbidity and LOS. These findings support the use of RARC as a standard approach to radical cystectomy, including in obese populations.

**Supplementary Information:**

The online version contains supplementary material available at 10.1007/s11701-025-02939-5.

## Introduction

The adoption of minimally invasive techniques has transformed the landscape of major oncological surgery. Over the past two decades, robot-assisted surgery has seen rapid expansion across multiple specialties, driven by enhanced visualization, improved dexterity, and potential benefits for perioperative recovery. In gynaecologic, colorectal, and pancreatic surgery, robotic approaches have consistently demonstrated reduced blood loss, fewer wound complications, and shorter hospital stays, particularly in obese patients, albeit at the expense of longer operative times and higher costs [1–3]. These findings provide a strong rationale for extending robotic techniques to complex urological procedures such as radical cystectomy.

In urology, open radical cystectomy (ORC) has long been considered the standard of care for muscle-invasive bladder cancer. However, robot-assisted radical cystectomy (RARC) has gained increasing acceptance worldwide. In the United States, RARC adoption increased steadily from 2008 through at least 2019, with national datasets showing proportions exceeding 50% in contemporary cohorts, while in Europe adoption increased from 2% in 2006 to as high as 50% in some regions by 2018 [4, 5]. This diffusion has been particularly pronounced in academic and high-volume centers, supported by evidence suggesting comparable oncologic outcomes, including negative margin rates and lymph node yields, as well as similar functional results in terms of continence and sexual function [6, 7].

Despite these advances, obesity remains a procedural challenge for cystectomy. Across surgical disciplines, higher body mass index (BMI) is linked to longer operations and greater perioperative risk [8, 9]. In the robotic setting, technical constraints in obese patients, such as restricted working space, the need for steep Trendelenburg positioning, and access limitations can increase procedural complexity and prolong operating time; this has been shown in obese gynaecologic cohorts and is consistent with comparative evidence that RARC itself tends to be longer than ORC [10–12]. Implementation studies in visceral surgery have also reported higher morbidity at very high BMI thresholds and have emphasized learning-curve and team factors that can amplify difficulty in obese robotic cases [13]. At the same time, several surgical specialties have documented advantages of robotics in obese patients, including lower blood loss, fewer wound complications, and shorter hospitalization, indicating that these perioperative benefits may offset some of the technical drawbacks [10, 14, 15]. Of note, recent expert consensus highlights that, with growing surgical experience and advances in robotic platforms, these challenges can be effectively managed and that RARC achieves perioperative outcomes in obese patients at least equivalent to, and in selected aspects superior to, ORC [16].

Evidence specific to RARC in obese bladder cancer patients, however, remains limited and heterogeneous. While small cohort studies suggest that outcomes after RARC may not be inferior in overweight or obese patients compared to normal-weight cohorts [17, 18], robust analyses directly addressing the interaction of BMI with surgical modality and perioperative morbidity are lacking.

Given the global rise in obesity and the parallel diffusion of robotic surgery, clarifying whether RARC offers equal or superior safety in overweight patients is of major clinical importance. The Department of Urology at the University Hospital Schleswig-Holstein, Kiel, was among the first centers in Germany to implement RARC, and the procedure has since evolved into our institutional standard of care. This study provides real-world evidence from a high-volume tertiary care center, complementing controlled trial data and reflecting outcomes in routine clinical practice. We aimed to test the non-inferiority of RARC in overweight patients, focusing on perioperative outcomes, hospital stay, and complication patterns, thereby addressing a critical evidence gap at the intersection of surgical innovation and patient comorbidities.

## Materials and methods

### Study design and patient cohort

This was a retrospective, single-center cohort study conducted at the Department of Urology, University Hospital Schleswig-Holstein (UKSH), Kiel, Germany. All consecutive patients who underwent radical cystectomy with urinary diversion for bladder cancer between August 2013 and December 2024 were eligible. Patients with non-urothelial histologies or non-malignant indications were excluded. The study population was stratified according to surgical approach [open radical cystectomy (ORC) vs. robot-assisted radical cystectomy (RARC)] and BMI (≤ 25 vs. >25 kg/m²).

### Ethical approval

The study was approved by the Ethics Committee of the Medical Faculty, Christian-Albrechts-University of Kiel (D 402/25). Data collection and analysis followed institutional and international ethical standards in accordance with the Declaration of Helsinki.

### Surgical technique and urinary diversion

ORC and RARC were performed according to institutional standards. RARC procedures were conducted using the da Vinci surgical system (Intuitive Surgical, Sunnyvale, CA, USA), utilizing the Si and Xi platforms during the study period (Si and Xi until 2022, thereafter Xi). Urinary diversions included non-continent procedures (ureterocutaneostomy, ileal conduit) and continent reconstructions (orthotopic neobladder and continent pouch). The choice of diversion was made based on patient characteristics and preference, tumor stage, and surgeon preference.

### Outcomes and variables

Baseline variables included age, sex, BMI, American Society of Anesthesiologists (ASA) score, Charlson Comorbidity Index (CCI), and tumor stage. Perioperative outcomes comprised type of urinary diversion (continent vs. non-continent), operative time (minutes), length of hospital stay (days), and postoperative complications within 90 days, classified according to the Clavien–Dindo system [19]. Complications were further categorized as infectious, cardiopulmonary, gastroenterological, anastomotic, or other.

The primary study objectives were to investigate (i) the influence of BMI and surgical approach on urinary diversion type, (ii) the impact of BMI and approach on operative time, (iii) the interaction between BMI and approach on hospital stay, (iv) the complication spectrum by BMI and approach, and (v) predictors of complication-free recovery and prolonged hospitalization.

### Statistical analysis

Categorical variables were summarized as frequencies and percentages, while continuous variables were expressed as mean ± standard deviation (SD) or median with interquartile range (IQR), as appropriate. Group comparisons were performed using the Chi² test or Fisher’s exact test for categorical data, and Student’s t-test or Mann–Whitney U test for continuous data, depending on distribution.

Regression analyses were applied to examine associations between BMI, surgical approach, and outcomes. Linear regression was used for operative time and hospital stay, stratified by surgical method and adjusted for age, ASA score, CCI, and tumor stage. Logistic regression was performed to assess predictors of urinary diversion type, complication-free recovery (Clavien–Dindo grade 0), and prolonged hospitalization (> 14 days), and results were reported as odds ratios (OR) with 95% confidence intervals (CI). Three-way interactions between surgical method, BMI, and complication type were investigated using log-linear modelling and multinomial logistic regression. Non-inferiority analyses for hospital stay and complication-free recovery in overweight patients were performed using a predefined non-inferiority margin of − 3 days for length of stay (RARC vs. ORC). This threshold reflects the smallest difference considered clinically important based on contemporary evidence that RARC typically shortens hospitalization by ~ 2–4 days compared with ORC; a difference exceeding 3 days against RARC would therefore represent a clinically unacceptable loss of benefit. We evaluated non-inferiority using two-sided 90% confidence intervals (equivalent to a one-sided α = 0.05); non-inferiority was concluded if the lower CI bound for Δ (RARC − ORC) exceeded − 3 days. For complication severity we prespecified a non-inferiority margin of − 1 Clavien–Dindo grade on the mean severity scale. Because transitions between adjacent grades (particularly II to III) reflect materially different clinical management, a shift of ≥ 1 grade toward higher severity was considered clinically unacceptable. As above, non-inferiority was evaluated using two-sided 90% CIs for Δ (RARC − ORC), concluding non-inferiority when the lower bound exceeded − 1 grade.

All analyses were conducted using R version 4.5.1 (2025-06-13, “Great Square Root”). A two-sided p-value < 0.05 was considered statistically significant. ChatGPT (OpenAI, San Francisco, CA, USA) was used exclusively for grammar, spelling, and language refinement.

## Results

### Patient cohort and baseline characteristics

**Fig. 1:**
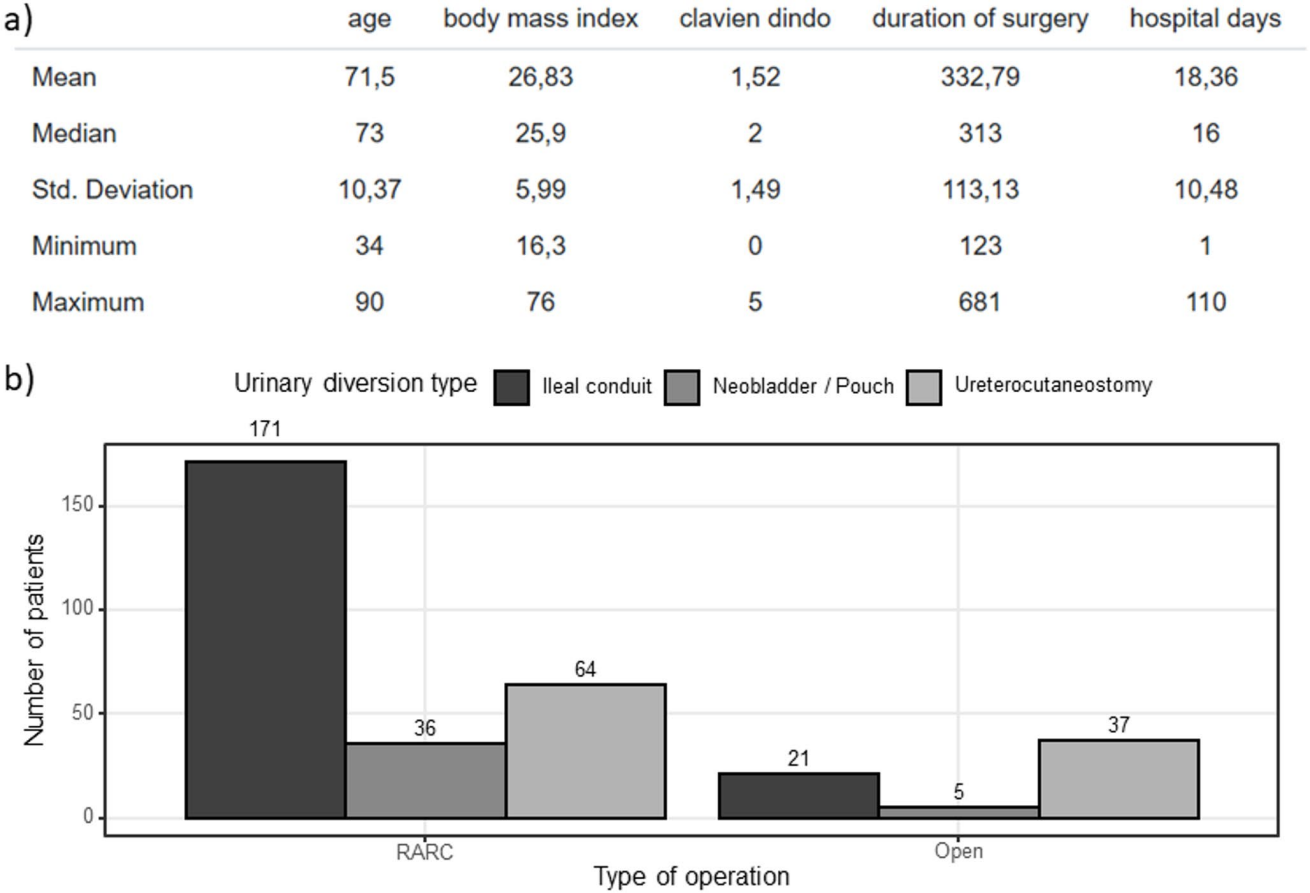
Patient cohort and baseline characteristics. (**a**) Summary statistics for the full cohort (*n* = 337), including age, body mass index (BMI), Clavien–Dindo complication grade, duration of surgery, and hospital stay. Values are presented as mean, median, standard deviation (SD), minimum, and maximum. (**b**) Urinary diversion by surgical approach: robot-assisted radical cystectomy (RARC; *n* = 271) and open radical cystectomy (ORC; *n* = 63). Bars represent counts with proportions as follows—after RARC: ileal conduit 171/271 (63.1%), neobladder/pouch 64/271 (23.6%), ureterocutaneostomy (UCN) 36/271 (13.3%); after ORC: ileal conduit 21/63 (33.3%), neobladder/pouch 5/63 (7.9%), UCN 37/63 (58.7%)

A total of 337 patients who underwent radical cystectomy were included in the analysis. The median age was 73 years (range 34–90), with a mean BMI of 26.8 kg/m² (range 16.3–76.0). Overall, the median Clavien–Dindo complication grade was 2 (mean 1.5, range 0–5). Median duration of surgery was 313 min (mean 332.8, range 123–681), and the median length of hospital stay was 16 days (mean 18.4, range 1–110) (see Fig. 1a).

Across the cohort, RARC was performed in 274 patients (81.7%), and ORC in 63 patients (18.3%). The distribution of urinary diversion types differed substantially between surgical approaches: after RARC, most patients received an ileal conduit (63.1%), followed by neobladder/pouch (23.6%) and ureterocutaneostomy (UCN; 13.3%). In contrast, after ORC, UCN was most frequent (58.7%), followed by ileal conduit (33.3%) and neobladder/pouch (7.9%) (see Fig. 1b).

When stratified by BMI, overweight patients (BMI > 25 kg/m²) were again more likely to receive an incontinent diversion compared to normal-weight patients (approx. 52% vs. 34%), while the overall rate of continent diversions remained low in both groups (see Online Resource 1, Fig. 1).

### Urinary diversion

When analyzing the relationship between BMI and surgical approach, incontinent urinary diversion was consistently more common in overweight patients, both in open and robotic procedures (Online Resource 1, Fig. 1). Continent diversions were performed less frequently in all subgroups.

In a binomial regression model with aggregated data, neither operative approach (RARC vs. ORC), BMI category (overweight vs. normal-weight), nor their interaction showed a significant effect on the likelihood of receiving a continent diversion (all *p* > 0.40; interaction term OR 2.34, 95% CI 0.26–17.74; Online Resource 1, Table 1). These findings suggest that the choice of diversion type was largely independent of BMI or surgical modality.

### Duration of surgery

**Fig. 2:**
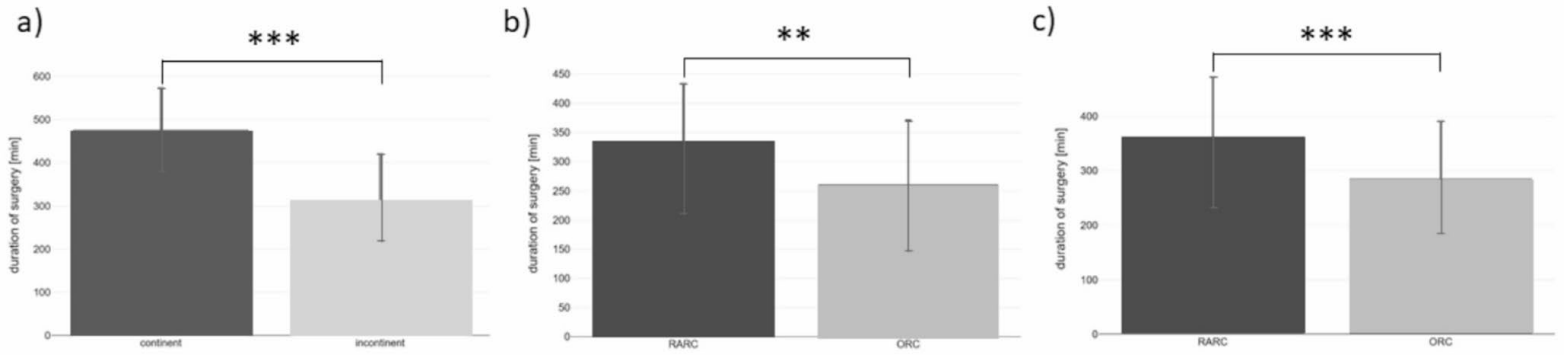
Duration of surgery by diversion type, surgical approach, and BMI subgroup. (**a**) Operative time according to urinary diversion: continent (orthotopic neobladder/pouch) vs. incontinent (ileal conduit or ureterocutaneostomy). Mean (± SD) times were 476.2 ± 96.5 vs. 310.7 ± 109.1 min, respectively (*p* < 0.001). (**b**) Normal-weight patients (BMI < 25 kg/m²): robot-assisted radical cystectomy (RARC; *n* = 96) vs. open radical cystectomy (ORC; *n* = 17). Mean operative time was 334.2 ± 99.3 vs. 261.4 ± 109.7 min (*p* = 0.004). (**c**) Overweight patients (BMI ≥ 25 kg/m²): RARC (*n* = 143) vs. ORC (*n* = 36). Mean operative time was 355.1 ± 117.2 vs. 285.4 ± 105.5 min (*p* < 0.001). Significance levels shown as * *p* < 0.05, ** *p* < 0.01 and *** *p* < 0.001

The mean operative time in the overall cohort was 332.8 min (SD 113.1; range 123–681; Fig. 1a). Stratification by type of urinary diversion revealed markedly longer operative times for continent compared to incontinent diversions (476.2 ± 96.5 vs. 310.7 ± 109.1 min, *p* < 0.001; Fig. 2a).

When analyzed by surgical approach, robot-assisted radical cystectomy (RARC) was consistently associated with longer operative times compared to open radical cystectomy (ORC). Among normal-weight patients (BMI < 25 kg/m²), mean operative time was significantly higher in RARC than ORC (334.2 ± 99.3 vs. 261.4 ± 109.7 min, *p* = 0.004; Fig. 2b). In overweight patients (BMI ≥ 25 kg/m²), RARC procedures were also significantly longer than ORC (355.1 ± 117.2 vs. 285.4 ± 105.5 min, *p* < 0.001; Fig. 2c).

To further assess the role of BMI, linear regression analyses were performed separately for both surgical approaches. BMI did not significantly influence operative time in ORC (B = 24.01, *p* = 0.41) or in RARC (B = 20.89, *p* = 0.15), suggesting that the observed differences in operative duration were mainly attributable to the surgical method rather than patient body mass.

### Hospital stay

**Fig. 3:**
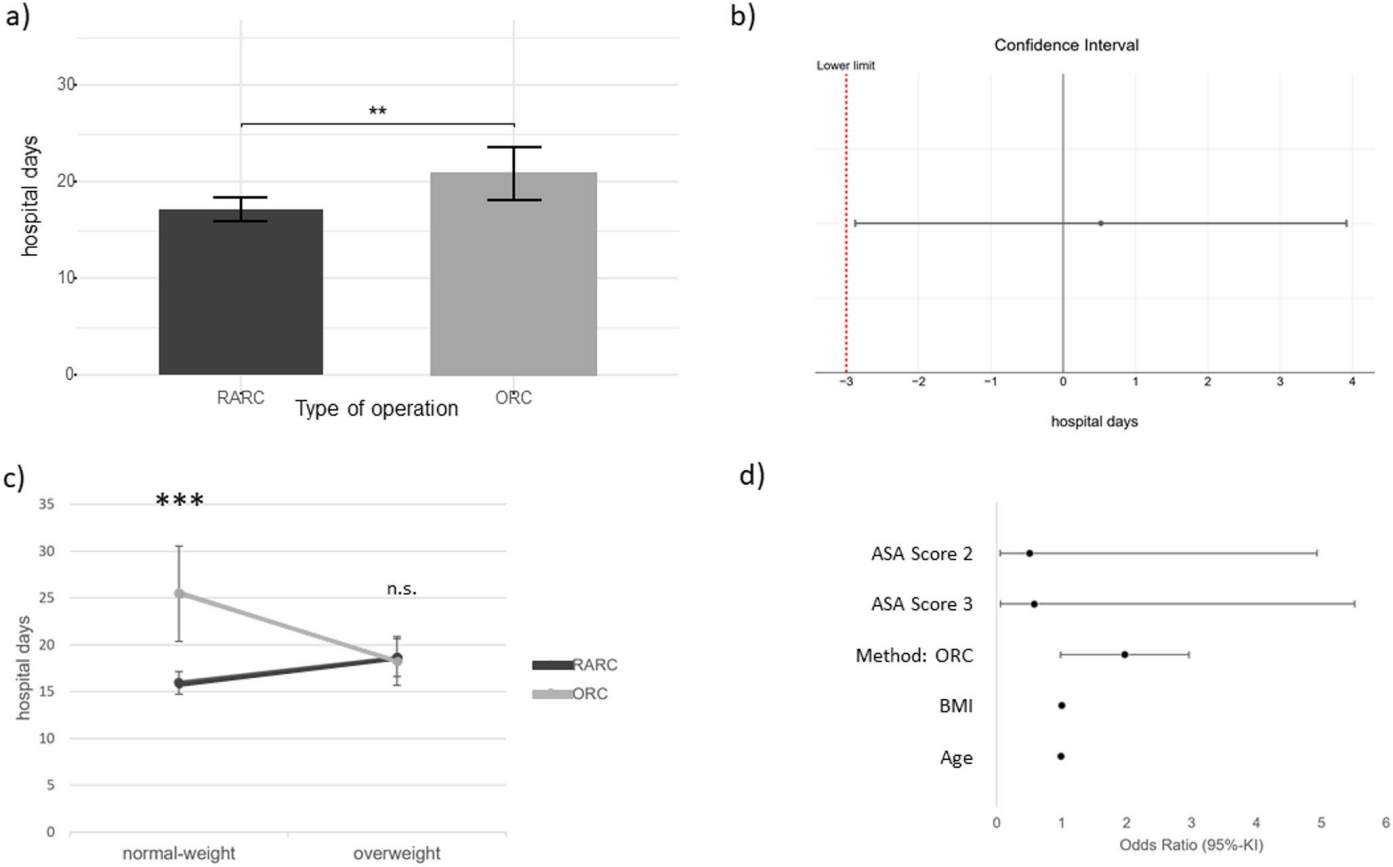
Length of hospital stay (LOS) by surgical approach, BMI subgroup, and multivariable analysis. (**a**) Overall cohort (*n* = 337): patients undergoing robot-assisted radical cystectomy (RARC) had a shorter LOS than those treated with open radical cystectomy (ORC) (17.6 ± 11.0 vs. 21.3 ± 10.2 days; *p* = 0.0017). (**b**) Non-inferiority (NI) analysis in overweight patients (BMI ≥ 25 kg/m²): mean difference Δ = +0.35 days (RARC–ORC) with two-sided 90% CI − 2.44 to + 3.14; the dashed vertical line marks the NI margin (− 3 days). Because the lower CI bound is above the margin, RARC is non-inferior to ORC for LOS. (**c**) LOS by BMI subgroup: in normal-weight patients (BMI < 25 kg/m²), ORC was longer than RARC (25.47 ± 10.06 vs. 15.95 ± 6.07 days; *p* < 0.001), whereas in overweight patients the difference was not significant (18.28 ± 8.15 vs. 18.63 ± 12.18 days; *p* = 0.84); method × BMI interaction *p* = 0.398. Points show means with SD. (**d**) Multivariable model for LOS (covariates: age, BMI, ASA score, surgical approach, tumor stage): ORC remained an independent predictor of longer LOS (B = 2.6; *p* = 0.045), while BMI was not significant. Significance levels shown as * *p* < 0.05, ** *p* < 0.01 and *** *p* < 0.001

In the overall cohort, the mean length of stay was 18.4 ± 10.5 days (Fig. 1a). Across surgical approaches, patients undergoing RARC had a significantly shorter hospitalization than those treated with ORC (17.6 ± 11.0 vs. 21.3 ± 10.2 days, *p* = 0.014; Fig. 3a).

Stratification by BMI revealed a distinct pattern. Among normal-weight patients, ORC was associated with markedly longer stays than RARC (15.95 ± 6.07 vs. 25.47 ± 10.06 days *p* < 0.001), whereas overweight patients showed no relevant difference between methods (18.3 ± 6.2 vs. 18.6 ± 12.2 days; *p* = 0.84). In normal‑weight patients (RARC *n* = 96; ORC *n* = 17), the mean difference in length of stay was Δ = −9.52 days (RARC–ORC; 90% CI − 13.66 to − 5.38), favoring RARC. In overweight patients (RARC *n* = 143; ORC *n* = 36), the mean difference was Δ = +0.35 days (RARC–ORC; 90% CI − 2.44 to + 3.14). The method × BMI interaction term did not reach statistical significance (*p* = 0.398; Fig. 3c).

In the adjusted regression model (including age, ASA score and BMI), ORC remained an independent predictor of prolonged hospitalization (B = 2.6, *p* = 0.045, Fig. 3d), whereas BMI was not an independent risk factor. The model explained only a minor proportion of variance in hospital stay (adjusted R² = 0.02; Online Resource 1, Fig. 4).

**Fig. 4:**
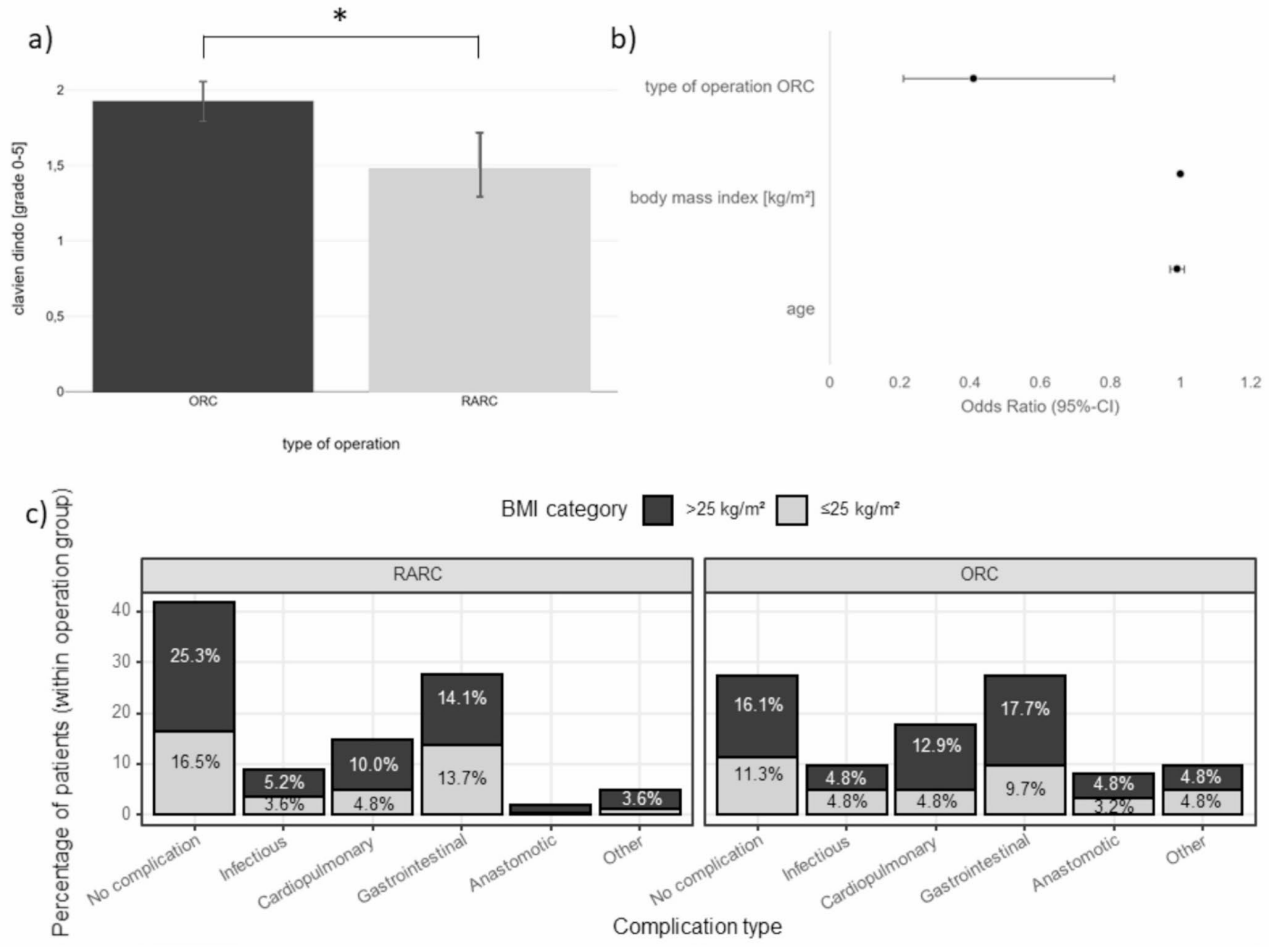
Postoperative complications by surgical approach and BMI. (**a**) Mean Clavien–Dindo severity in overweight patients (BMI ≥ 25 kg/m²) by approach: open radical cystectomy (ORC) vs. robot-assisted radical cystectomy (RARC). Severity was higher after ORC (1.93 ± 0.13) than after RARC (1.48 ± 0.24; *p* < 0.014); the mean difference was Δ = −0.45 grades (RARC–ORC; two-sided 90% CI − 0.90 to 0.00). (**b**) Multivariable analysis (forest plot) depicting odds ratios (95% CI) for key predictors of postoperative outcome: ORC was associated with worse outcomes (lower odds of an uncomplicated course/higher complication severity), whereas BMI and age showed no significant associations. (**c**) Spectrum of complications after ORC and RARC stratified by BMI (normal-weight vs. overweight): cardiopulmonary and anastomotic events predominated in ORC, with higher counts among overweight patients. A higher proportion of complication-free courses is observed in both BMI groups in RARC, although cardiopulmonary events are relatively more frequent in overweight patients

Finally, in the prespecified non‑inferiority analysis for overweight patients, the mean difference in LOS was + 0.35 days (RARC–ORC; 90% CI − 2.44 to + 3.14); because the lower bound (− 2.44) exceeded the − 3‑day margin, RARC met the criterion for non‑inferiority (Fig. 3b).

### Complications

In the analysis of postoperative complications, overweight patients (BMI > 25 kg/m²) undergoing ORC had significantly higher mean Clavien-Dindo grades compared to those after RARC (1.93 ± 0.13 vs. 1.48 ± 0.24; *p* = 0.014; Cohen’s d = 0.29; mean difference − 0.45 grades (RARC − ORC); 90% CI of − 0.90 to 0.00; Fig. 4a). The likelihood of remaining complication-free was also significantly lower after ORC (OR = 0.47, 95% CI: 0.25–0.88) compared to RARC (OR = 2.12, 95% CI: 1.14–3.95; Online Resource 1, Fig. 3).

When stratified by BMI and type of surgery, distinct complication spectra emerged. Among patients undergoing ORC, cardiopulmonary and gastroenterological complications predominated, with overweight patients showing higher frequencies (Fig. 4c). In contrast, RARC patients, regardless of BMI, showed higher proportions of complication-free outcomes, though overweight patients more often experienced cardiopulmonary complications (Fig. 4d).

The non-inferiority analysis confirmed that RARC in overweight patients was not inferior to ORC regarding postoperative outcome. The lower boundary of the 90% confidence interval (− 0.88) remained above the predefined non-inferiority margin of − 1 Clavien-Dindo grade (*p* = 0.044; Online Resource 1, Fig. 4).

Regression analysis supported these findings: ORC remained an independent predictor of higher complication severity, while BMI alone was not significant (Fig. 4b).

In a detailed spectrum analysis (Online Resource 1, Table 2), significant differences in complication patterns were observed between groups (χ²(15) = 38.7, *p* = 0.0007). A log-linear model confirmed a significant three-way interaction between method, BMI, and complication type (ΔDev = 13.38, df = 5, *p* = 0.020). Multinomial regression indicated a specific signal for cardiopulmonary complications: overweight patients after RARC had significantly higher odds compared to ORC (*p* = 0.040). No significant BMI-related interactions were found for gastrointestinal complications, complication-free courses, or other categories.

## Discussion

In this retrospective, single-center cohort study, we provide real-world evidence from a high-volume European tertiary care center that robot-assisted radical cystectomy (RARC) is non-inferior to open radical cystectomy (ORC) in overweight patients with respect to perioperative outcomes, length of hospital stay, and complication profiles. While RARC was consistently associated with longer operative times, body-mass index (BMI) itself did not significantly influence operative duration within either approach. Importantly, the probability of remaining complication-free was preserved in overweight patients undergoing RARC, and non-inferiority for hospital stay was statistically confirmed. These findings expand current evidence by demonstrating that RARC maintains safety across BMI subgroups under real-world conditions, complementing data from controlled trials and clarifying an evidence gap for this common comorbidity.

The adoption of RARC has steadily increased worldwide over the past two decades.

In the United States, its use rose from < 1% in 2008 to over 20% by 2013, and in Europe from 2% in 2006 to nearly 50% by 2018 [20, 21].

This diffusion has been driven by advantages such as reduced blood loss, lower transfusion rates, shorter hospitalization, and comparable or superior lymph node yields compared with ORC [22–24].

Our institution was among the first in Germany to implement RARC, and it has since evolved into our standard approach for radical cystectomy. By reporting a decade-long, unselected real-world cohort, our study complements controlled trials and situates robotic cystectomy within routine clinical practice.

### Urinary diversion

BMI did not significantly influence the choice of urinary diversion in either RARC or ORC.

This contrasts with earlier reports that suggested surgeons often favored non-continent diversions in obese patients because of perceived technical complexity [25].

Our data therefore support that, in experienced centers, BMI alone should not limit access to continent reconstructions in either surgical approach. This finding is in line with more recent institutional series and underscores that surgeon expertise and institutional workflow rather than patient BMI determine diversion selection.

### Duration of surgery

As widely reported, RARC required longer operative times than ORC in both normal-weight and overweight patients [21, 24]. In our cohort, mean operative times were significantly prolonged for RARC (334 vs. 261 min in normal-weight, 355 vs. 285 min in overweight; all *p* < 0.01). However, regression analyses confirmed that BMI itself did not significantly prolong operative time within either technique (B = 24.0 for ORC, *p* = 0.41; B = 20.9 for RARC, *p* = 0.15), a finding that contrasts with reports from gynecologic or colorectal robotic surgery where obesity more strongly increased procedural duration [26, 27]. These differences likely reflect distinct anatomical workspaces and the long robotic experience at our center. Taken together, our results suggest that the choice of surgical approach rather than BMI drives operative time in contemporary cystectomy practice.

### Hospital stay

Length of hospital stay is a key patient-centered outcome and has often favored RARC.

In our unadjusted analysis, ORC patients who were normal-weight had significantly longer stays than their overweight counterparts (25.5 ± 10.1 vs. 18.3 ± 6.2 days, *p* = 0.0017), whereas in RARC no significant difference was seen between normal-weight and overweight patients (15.4 ± 6.1 vs. 18.6 ± 12.2 days, *p* = 0.398).

Multivariate models confirmed ORC (but not BMI) as an independent predictor of prolonged hospitalization (B = 2.6, *p* = 0.045), and non-inferiority testing in overweight patients met the prespecified margin (lower 90% CI − 2.88 days above − 3 days; *p* = 0.044). Prior studies have reported shorter hospital stays with RARC, attributed to less blood loss, smaller incisions, and faster mobilization [21, 22]. Contemporary U.S. cohorts and randomized evidence indicate that RARC yields modestly shorter LOS and markedly lower transfusion rates, with similar rates of major (≥ III) complications and 30-day readmission compared with ORC [11].

Our findings confirm these benefits in a real-world cohort and highlight that robotic surgery dampens BMI-related variability in hospitalization observed with ORC. Our absolutes LOS is longer than the reported by contemporary U.S. enhanced recovery after surgery (ERAS) centers where median LOS typically ranges from 5 to 9 days post-implementation (vs. 8–12 days pre-implementation). These differences may likely reflect system-level differences, such as discharge norms, rehabilitation availability and regulatory environments rather than intrinsic differences in surgical safety [28, 29].

### Complications

Overall complication rates after cystectomy remain high irrespective of approach. Our findings are consistent with prior reports showing broadly similar complication rates for RARC and ORC, with a tendency toward fewer low-grade complications in robotic series [23, 24]. Three-way log-linear analysis demonstrated a significant interaction between surgical method, BMI, and complication type (*p* = 0.020), indicating that BMI influences the complication pattern differently in RARC versus ORC. Specifically, overweight patients undergoing RARC experienced more cardiopulmonary complications (interaction *p* = 0.040), consistent with literature citing increased pulmonary risk in obese patients exposed to steep Trendelenburg positioning during robotic pelvic surgery [26].

Nevertheless, the overall likelihood of remaining complication-free was not adversely affected by BMI, and RARC achieved higher rates of complication-free recovery across BMI strata.

These data refine understanding of perioperative risk by identifying a specific vulnerability, cardiopulmonary events in overweight RARC patients, while confirming the overall safety of the robotic approach.

### Strengths and limitations

This study has several strengths. It reports one of the largest single-center series from Europe with long-term robotic cystectomy experience, incorporating over a decade of practice. The integration of multivariate models and three-way interaction analyses provides a nuanced assessment of how BMI and surgical method jointly affect perioperative outcomes, a level of granularity rarely addressed in previous reports. Furthermore, the study reflects real-world outcomes from a high-volume tertiary center, thereby enhancing external validity.

Limitations include the retrospective design and single-center setting, which may limit generalizability. Despite the relatively large overall sample, subgroup analyses occasionally suffered from small cell counts, particularly in specific complication categories, necessitating cautious interpretation of Chi² results. Additionally, long-term oncological outcomes and functional results (e.g., continence, sexual function) were not addressed here but have been shown in prior studies to be comparable between ORC and RARC [22, 23].

### Future directions

Future research should build on these findings with multicenter, prospective studies focusing on obese and high-risk populations. Integration of cost-effectiveness analyses, patient-reported outcomes, and long-term oncological endpoints will be crucial to fully establish the role of RARC in this subgroup. Enhanced recovery protocols specifically tailored for overweight patients undergoing robotic surgery may further mitigate cardiopulmonary risks and optimize outcomes.

## Conclusion

In summary, our study demonstrates that RARC is non-inferior to ORC in overweight patients with regard to perioperative safety, hospital stay, and complication patterns. These findings support the use of robotic surgery as a standard approach to radical cystectomy, including in patients with elevated BMI, and provide robust real-world evidence from a high-volume European center.

## Supplementary Information

Below is the link to the electronic supplementary material.

Supplementary Material 1

## Data Availability

De-identified data and analysis code are available from the corresponding author on reasonable request; data sharing is subject to institutional and legal restrictions.

## Declarations

### Use of generative AI and AI-assisted technologies

Language polishing was assisted by ChatGPT (OpenAI) for grammar/clarity only. No AI tool was used to generate, analyze, or interpret data. All authors reviewed and take full responsibility for the content.
