# Supplementary material for: Expanding the role of robotic surgery: Non-inferiority of robot-assisted radical cystectomy in overweight patients with bladder cancer
Source: J Robot Surg. 2025 Nov 12;20(1):9. doi: 10.1007/s11701-025-02939-5 (PMC12605525; doi:10.1007/s11701-025-02939-5)
Supplement: Supplementary file 1 — Supplementary Material 1 [file 11701_2025_2939_MOESM1_ESM.pdf]

Online Resources:

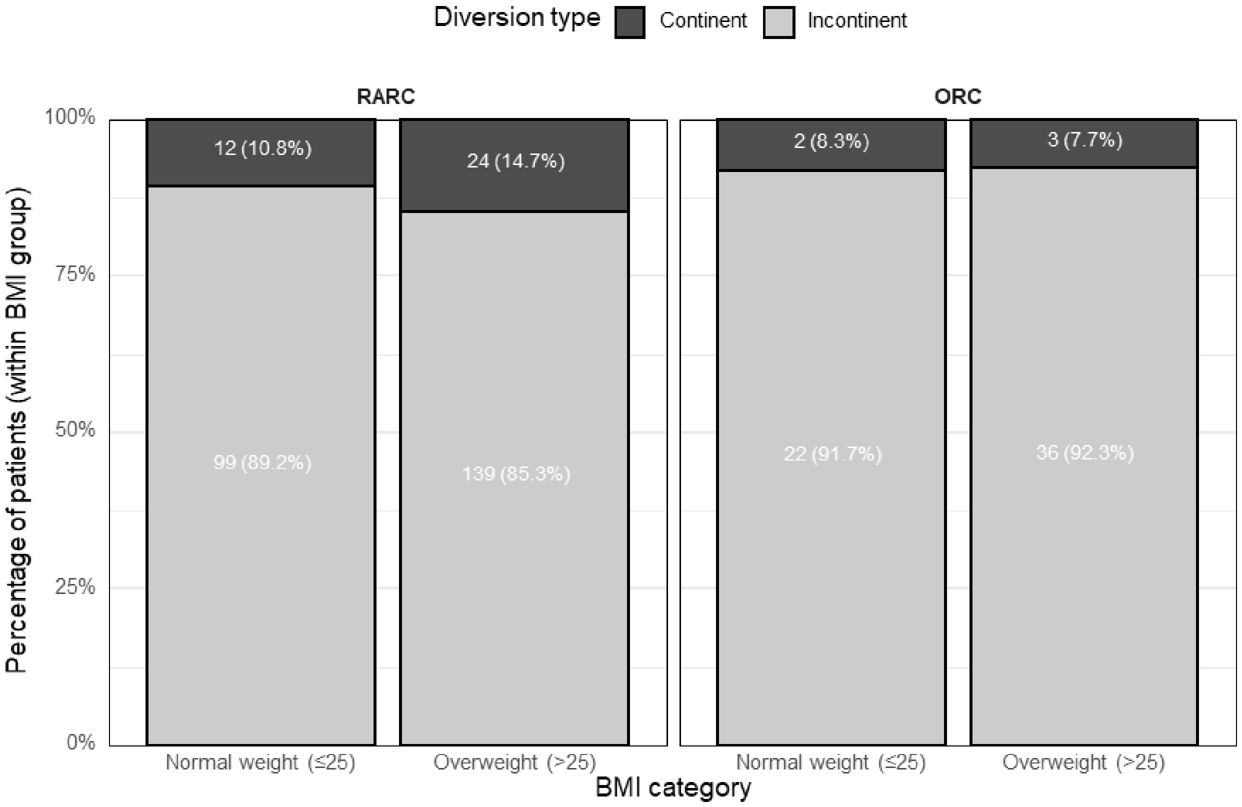

Fig. 1 Type of urinary diversion by BMI category and surgical method. Distribution of continent and incontinent urinary diversion types stratified by body mass index (BMI) category and surgical approach. Population: n = 334. Groups: normal weight (≤25 kg/m²) and overweight (>25 kg/m²); surgical method: robot-assisted radical cystectomy (RARC) and open radical cystectomy (ORC). Bars represent proportions of continent (dark grey) and incontinent (light grey) diversions within each subgroup, shown as absolute counts and percentages. After RARC, continent diversions were performed in 12/111 (10.8%) normal-weight and 24/163 (14.7%) overweight patients; after ORC, continent diversions were performed in 2/24 (8.3%) and 3/39 (7.7%), respectively.

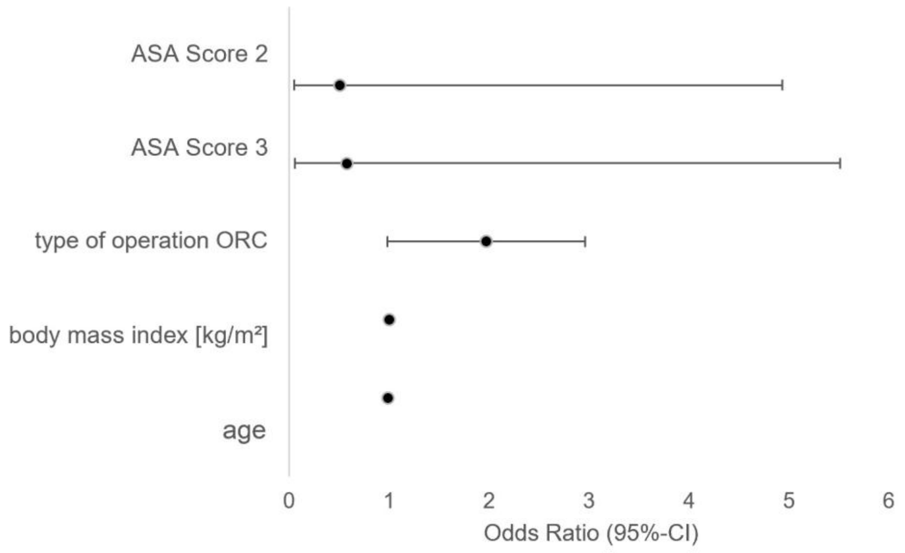

Fig. 2 Risk factors for prolonged hospital stay (>14 days) after radical cystectomy. Forest plot from a multivariable logistic regression showing adjusted odds ratios (OR) with 95% confidence intervals (CI) for ASA class, type of operation (ORC vs RARC), body mass index (BMI), and age in the study cohort. Points denote adjusted ORs and horizontal bars the 95% CI; the reference line is OR = 1. Reference categories and scaling: ASA 1 (reference, not plotted); operation type reference = RARC; BMI per 1 kg/m²; age per 1 year. Abbreviations: ASA, American Society of Anesthesiologists; ORC, open radical cystectomy; RARC, robot-assisted radical cystectomy; CI, confidence interval; BMI, body-mass index

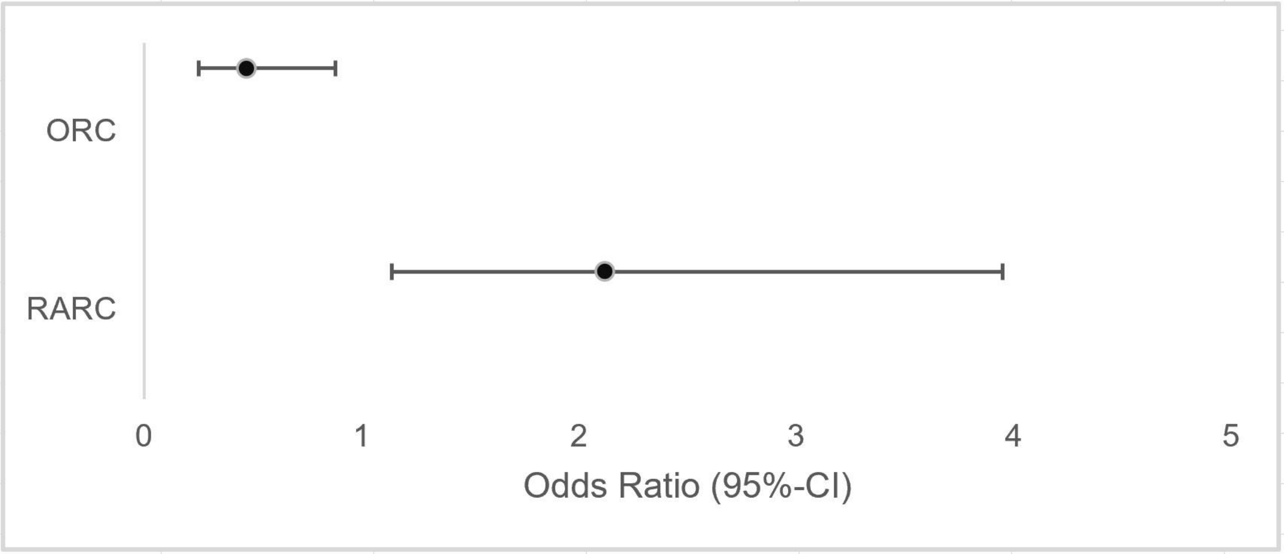

Fig. 3 Odds of a complication-free postoperative course by surgical approach. Points show odds ratios (OR) and horizontal bars 95% confidence intervals (CI) from logistic regression in the study cohort (n = 307); estimates: ORC 0.47 (95% CI 0.25–0.88) and RARC 2.12 (95% CI 1.14–3.98). Abbreviations: ORC, open radical cystectomy; RARC, robot-assisted radical cystectomy; CI, confidence interval

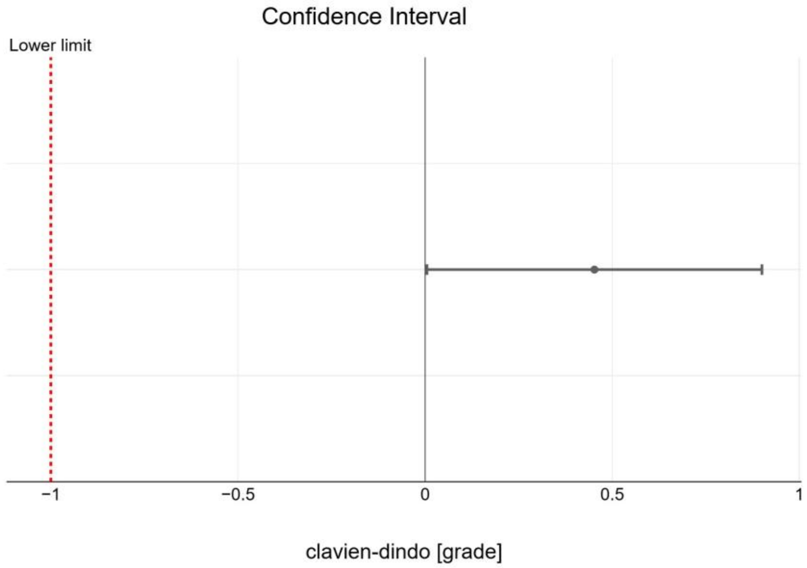

Fig. 4 Non-inferiority of postoperative morbidity in overweight patients (BMI > 25 kg/m²) comparing RARC with ORC. Point estimate and 95% confidence interval (CI) for the difference in Clavien–Dindo grade (RARC − ORC); the vertical dashed line at −1 denotes the prespecified non-inferiority margin (maximum tolerable difference of 1 grade). The 95% CI lies entirely to the right of this margin, indicating that RARC is not worse than ORC within a one-grade tolerance on the Clavien–Dindo scale. Abbreviations: RARC, robot-assisted radical cystectomy; ORC, open radical cystectomy; CI, confidence interval; BMI, body-mass index

Tab. 1 Logistic regression for the odds of receiving a continent urinary diversion by surgical approach and BMI, including the method × BMI interaction. Entries are odds ratios (OR) with 95% confidence intervals (CI) and p-values; OR > 1 indicates higher odds of continent diversion. Reference categories: surgical method ORC (vs RARC) and BMI ≤ 25 kg/m² (vs > 25 kg/m²). Abbreviations: OR, odds ratio; CI, confidence interval; BMI, body-mass index; ORC, open radical cystectomy; RARC, robot-assisted radical cystectomy

Logistic regression: continent diversion ~ method * BMI				
Term	OR	95% CI low	95% CI high	p
(Intercept)	0.1111111	0.01765998	0.3848172	0.003199549
methodRARC	1.1379310	0.27418323	7.7588913	0.873436339
bmioverweight	0.6923077	0.10570636	5.6006767	0.700589791

methodRARC:bmio-verweight	2.3410333	0.25803437	17.7408910	0.409839310

Tab. 2: Complication spectrum after radical cystectomy by surgical approach and BMI category. Counts (n) and percent within method are shown for each complication type (none, infectious, cardiopulmonary, gastroenterological, etc.); percentages are calculated within the corresponding surgical method subgroup and may not sum to 100 due to rounding. Abbreviations: ORC, open radical cystectomy; RARC, robot-assisted radical cystectomy; BMI, body-mass index (normal-weight ≤25 kg/m²; overweight >25 kg/m²)

Complication spectrum by method, BMI and type				
Method	BMI	Complication type	n	% within method
ORC	overweight	none	11	17.7
ORC	normal	none	5	8.1
RARC	overweight	none	62	25.5
RARC	normal	none	41	16.9
ORC	overweight	infectious	3	4.8
ORC	normal	infectious	3	4.8
RARC	overweight	infectious	14	5.8
RARC	normal	infectious	8	3.3
ORC	overweight	cardiopulmonary	3	4.8
ORC	normal	cardiopulmonary	9	14.5
RARC	overweight	cardiopulmonary	29	11.9
RARC	normal	cardiopulmonary	6	2.5
ORC	overweight	gastroenterological	5	8.1
ORC	normal	gastroenterological	12	19.4

RARC	overweight	gastroenterological	43	17.7
RARC	normal	gastroenterological	26	10.7
ORC	overweight	anastomotic	4	6.5
ORC	normal	anastomotic	1	1.6
RARC	overweight	anastomotic	1	0.4
RARC	normal	anastomotic	1	0.4
ORC	overweight	other	3	4.8
ORC	normal	other	3	4.8
RARC	overweight	other	9	3.7
RARC	normal	other	3	1.2
